# Electrocatalytic Oxidation of Ascorbic Acid Using a Poly(aniline-*co*-*m*-ferrocenylaniline) Modified Glassy Carbon Electrode

**DOI:** 10.3390/s111110166

**Published:** 2011-10-26

**Authors:** Sanoe Chairam, Worawit Sriraksa, Maliwan Amatatongchai, Ekasith Somsook

**Affiliations:** 1 Department of Chemistry and Centre for Innovation in Chemistry, Faculty of Science, Ubon Ratchathani University, Warinchamrap, Ubon Ratchathani 34190, Thailand; E-Mails: lolekcity@hotmail.com (W.S.); m_amatatongchai@yahoo.com (M.A.); 2 NANOCAST Laboratory, Centre for Catalysis, Department of Chemistry and Centre for Innovation in Chemistry, Faculty of Science, Mahidol University, Rama 6 Road, Bangkok 10400, Thailand; E-Mail: nanocast@live.com (E.S.)

**Keywords:** electrochemical copolymerization, poly(aniline-*co*-*m*-ferrocenylaniline), electrocatalytic oxidation, ascorbic acid (AA)

## Abstract

A poly(aniline-*co-m*-ferrocenylaniline) was successfully synthesized on a glassy carbon electrode (GCE) by electrochemical copolymerization using a scan potential range from −0.3 to +0.9 V (*vs.* Ag/AgCl) in 0.5 M H_2_SO_4_ containing 30% acetonitrile (ACN), 0.1 M aniline (Ani) and 0.005 M *m*-ferrocenyaniline (*m*-FcAni). The field emission scanning electron microscope (FESEM) and electrochemical methods were used to characterize the poly(Ani-*co*-*m*-FcAni) modified electrode. The poly(Ani-*co*-*m*-FcAni)/GCE exhibited excellent electrocatalytic oxidation of ascorbic acid (AA) in citrate buffer solution (CBS, pH 5.0). The anodic peak potential of AA was shifted from +0.55 V at the bare GCE to +0.25 V at the poly(Ani-*co*-*m*-FcAni)/GCE with higher current responses than those seen on the bare GCE. The scan number at the 10th cycle was selected as the maximum scan cycle in electrochemical polymerization. The limit of detection (LOD) was estimated to be 2.0 μM based on the signal-to-noise ratio (S/N = 3). The amperometric responses demonstrated an excellent selectivity for AA determination over glucose (Glu) and dopamine (DA).

## 1. Introduction

l-Ascorbic acid (AA, vitamin C) is the major antioxidant found in many plants. As known, AA is an essential nutrient that has been widely used on a large scale as an antioxidant agent in foods, beverages and pharmaceutical applications, due to its participation in several human metabolic reactions [1]. The analytical determination of AA has been reported by many methodologies, such as enzymatic methods [2], iodometric titration using 2,6-dichlorophenol-indophenol as indicator [3], spectroscopy [4], chromatography [5], fluorimetry [6] and electrochemistry [7,8]. Due to their quick response, high sensitivity, low detection limit and simple use, electrochemical methods are currently of much interest for AA determination by the electrocatalytic oxidation reaction on conventional electrodes. Though AA is an important antioxidant compound, it is difficult to determine by direct oxidation on conventional electrodes because of interfering species such as dopamine (DA) and glucose (Glu) [8,9]. Thus, the development of electrodes for determination of AA in the presence of many interfering species has recently attracted much attention in the field of electroanalytical chemistry.

Electrodeposition of the conducting polymer film at the surface of an electrode is a modern approach that has been utilized extensively in a field of electrochemistry to modify electrodes for determination of AA [10–18]. Consequently, applications of modified electrodes in electrocatalysis and sensors have been enriched by the specific properties of conducting polymers. These are e.g., polypyrrole, polyaniline and polythiophene. Their molecules contain conjugated systems which are the reason for electron mobility in the molecule. Among conductive electroactive polymers, polyaniline (PAni) [10–13] and its derivatives [19] have been the most intensively prepared and also studied due to their unique properties, which are also favorable for their potential applications, such as chemical sensors and biosensors. However the electrochemical activity and stability of PAni are generally affected by a variety of solution conditions, such as electrolytes, solvents and pH. For example, Mu [20,21] reported that PAni by itself reveals excellent redox functions only in acidic media, pH < 3, and this feature limits its broad use. Thus, the copolymerization of aniline with ring-substituted aniline derivatives has been studied in order to modify the desired properties of polyanilines. A different type of conductance exists in the redox polymers, where redox centres are inserted into the polymer. PAni containing many groups including alkyl (–R), alkoxy (–OR), hydroxyl (–OH), amino (–NH_2_) or halogens (–X) and the position of substituents is expected to lead to significantly different chemical and physical properties from the parent polymer.

The modified electrodes for chemical sensors are generally fabricated by incorporating various compounds such as biomolecular [22], organic [17] and organotransition-metal compounds [20,23–26], either by physical or covalent attachment to the polymeric structure, in order to create novel electrochemical properties. In the field of electrochemistry, ferrocene (Fc) [20,23] and its derivatives [25,26] have been widely incorporated into polymeric materials, due to their ability to display the high redox behavior of the ferrocene/ferricinium (Fc/Fc^+^) couple in organic and aqueous solvents, including ionic liquids. Ferrocene can be easily oxidized and reduced reversibly. Due to their chemical versatility with high thermal stability, ferrocene moieties have been employed in a variety of applications [27–30], but are the most commonly used as the electrochemical active species for chemically modified electrodes (CMEs) [31]. The main problem of ferrocene is its tendency to be washed out of the matrix gradually [22,32]. In addition, ferrocene may be toxic and pollute to environment, if used on a large scale in the synthetic process for preparation of modified electrodes. Thus, it would be useful if the ferrocene could also be covalently bonded to the matrix, which is used to modify the conventional electrodes in order to investigate novel properties as mentioned above. Thus, in the present work, a poly(Ani-*co*-*m*-FcAni)/GCE electrode was electrochemically synthesized by using cyclic voltammetry. To explore its potential applications, the prepared electrode was used as a chemical sensor for the electrocatalytic oxidation of AA.

## 2. Experimental Section

### 2.1. Chemicals and Solutions

Aniline (Ani), l-ascorbic acid (AA), ethanol (EtOH), acetonitrile (ACN), sulfuric acid (H_2_SO_4_), sodium hydroxide (NaOH) and hydrochloric acid (HCl) were purchased from Sigma-Aldrich. All chemicals used in this study were of AR grade and used as received, except for the aniline, which was purified by double distillation under reduced pressure prior to use, and stored at 4 °C in refrigerator when not in use. All aqueous solutions were freshly prepared using de-ionized (DI) water (R ≥ 18.2 MΩ cm) purified with a Nanopore ultrapure water system. A 1,000 ppm stock standard AA solution was prepared freshly each day. The citrate buffer solution (CBS) (pH 5.0) was prepared by mixing 0.1 M trisodium citrate and 0.1 M citric acid.

### 2.2. Synthesis of *m*-FcAni

The *m*-FcAni monomer was synthesized from *m*-nitroaniline by following a method described in detail elsewhere [33,34]. After the solvent was removed, the crude product was absorbed onto silica and then purified by column chromatography with gradient elution (hexane-ethyl acetate) to afford the ferrocene derivative. A yellow-orange crystalline solid was obtained after drying under reduced pressure at room temperature.

### 2.3. Electrochemical Copolymerization of Poly(Ani-*co-m*-FcAni)

The GCE was polished carefully with alumina (Al_2_O_3_) slurry (1.0, 0.3 and 0.05 μm, respectively) using a soft polishing cloth, then thoroughly rinsed several times with DI water. After that, the GCE was sonicated in DI water for 10 min to remove alumina adsorbed on the electrode surface. The GCE was cleaned by potential cycling between −1.0 V and +1.0 V (*vs.* Ag/AgCl) at 50 mV s^−1^ in 0.1 M H_2_SO_4_ until a stable clean GCE cyclic voltammogram (CV) was obtained. The poly(Ani-*co*-*m*-FcAni) was successfully copolymerized electrochemically on the GCE surface using a scan potential ranging from −0.3 V to +0.9 V (*vs.* Ag/AgCl) in 0.5 M H_2_SO_4_ containing 30% ACN, 0.1 M Ani and 0.005 M *m*-FcAni. A thin film of poly(Ani-*co*-*m*-FcAni) coated on the GCE was thus obtained. Then, the poly(Ani-*co*-*m*-FcAni)/GCE was washed with 0.1 M H_2_SO_4_. EtOH and DI water to remove unreacted monomers from the electrode surface, and dried in air at room temperature (RT, 25 °C) for 1 h. The poly(Ani-*co*-*m*-FcAni)/GCE was kept in 0.1 M CBS (pH 5.0) at 4 °C in the fridge when not in use.

### 2.4. Instruments and Measurement Set Up

#### Cyclic Voltammetry

All cyclic voltammetric and amperometric experiments were performed using an AUTOLAB (PGSTAT-12) electrochemical analyzer (Metrohm, Switzerland) controlled by the GPES 4.9 software. A conventional three-electrode system was used throughout. The working electrode was the bare GCE (Φ = 3.0 mm) or the poly(Ani-*co-m*-FcAni)/GCE. All potentials were reported *versus* Ag/AgCl (sat. 3.0 M KCl) reference electrode. A platinum (Pt) wire was employed as the counter electrode. The reaction cell volume of 10 cm^3^ was used for all electrochemical measurements at RT. Measurements were carried out in 0.1 M CBS (pH 5.0) used as supporting-electrolyte solution. The pH of buffer solutions was monitored by using a 713 pH meter (Metrohm, Switzerland).

#### Hydrodynamic Voltammetry

Amperometric measurements at the poly(Ani-*co-m*-FcAni)/GCE were carried out at the potential of +0.25 V (*vs.* Ag/AgCl). The amperometric response of AA was shown in the amperogram. The current steps were associated with successive additions of 20 μL of 0.1 M AA standard solution into a stirred batch system using a 10 mL volume glass cell.

#### Electron Microscopy

The SEM images were recorded employing a JEOL JSM-5910 field emission scanning electron microscope (FESEM) by accelerating at a voltage of 15.0 kV. The surface of the poly(Ani-*co-m*-FcAni)/GCE was analyzed by mounting the sample onto a double-sided carbon tape, and then gold sputter coating to minimize charging prior to SEM imaging.

## 3. Results and Discussion

### 3.1. Characteristics of Poly(Ani-*co-m*-Fcani)

**Figure 1.**
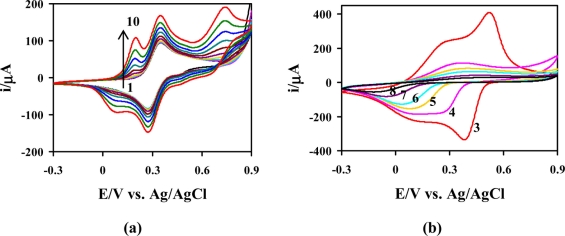
**(a)** The film growth of the poly(Ani-*co*-*m*-FcAni) during electrolysis of 0.1 M Ani, and 0.005 M *m*-FcAni in 0.5 M H_2_SO_4_ containing 30% ACN: (1) 1st cycle and (10) 10th cycle at the scan rate of 50 mV s^−1^. **(b)** The CVs of the poly(Ani-*co*-*m*-FcAni) modified electrode at different pH at 3, 4, 5, 6, 7 and 8 at a scan rate of 50 mV s^−1^.

The potential was ranged from −0.3 V to +0.9 V (*vs.* Ag/AgCl) at a scan rate of 50 mV s^−1^. This condition was also used to investigate the electrochemistry of aniline and its derivatives in an acid medium. Figure 1(a) shows the CVs of 0.1 M Ani and 0.005 M *m*-FcAni in 0.5 M H_2_SO_4_ containing 30% ACN. From the 1st-cycle to the 4th-cycle, a couple of reversible redox peaks (*E*_p,a_ = +0.35 V and *E*_p,c_ = +0.27 V) were observed as the iron center of the ferrocene units interconvert between the Fe^2+^ and Fe^3+^ states [29,31,35]. The inductive and steric effects of the ferrocene moieties make the monomer less reactive and difficult for the polymerization reaction. As the cycling process continued, two pairs of redox peaks on CVs located at +0.21/+0.09 V and +0.73/+0.67 V were observed from the 4th-cycle to the 10th-cycle. The peak currents increased gradually with increasing scan number, indicating an autocatalytic polymerization which causes the polyaniline film growth as the electrolysis proceeds [36]. After rinsing the working electrode with 0.1 M H_2_SO_4_. EtOH and DI water, respectively, a green-brown thin film was seen on the GCE surface, indicating that the poly(Ani-*co*-*m*-FcAni) was successfully copolymerized electrochemically.

**Scheme 1.**
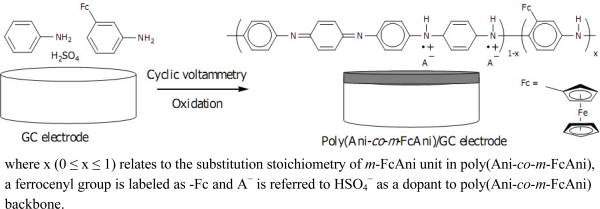
The electrochemical copolymerization of the conducting poly(Ani-*co*-*m*-FcAni) film modified GCE.

Figure 1(b) shows the responses of the poly(Ani-*co*-*m*-FcAni) modified electrode at different pH values. Strong anode and cathode peaks were observed in low pH solutions (pH < 4), indicating an excellent redox activity under acidic conditions. With increasing pH values, the redox peaks moved closer. This redox process leads to the dependence on the pH of solutions, the reactions and the polymer states [37]. In solutions of pH greater than 4, the poly(Ani-*co*-*m*-FcAni) nearly loses its electrochemical activity entirely, which corresponds to the leucoemeraldine/pernigraniline reaction. The electrochemical behavior of the poly(Ani-*co*-*m*-FcAni), according to the cyclic voltammogram, was similar to that the pure polyaniline and its derivatives in different solutions [19,20,38]. The electrochemical copolymerization of the conducting poly(Ani-*co*-*m*-FcAni) film modified GCE is illustrated in Scheme 1.

Based on our results, the proposed mechanism was initiated by the formation and reactions of cation radicals and dimeric species. The *m*-FcAni monomer was oxidized to generate the ferricinium species of *m*-FcAni^+•^. The formation of the radical cation generated from *m*-FcAni by electro-oxidation on the GCE surface was considered as the rate-determining step. This was followed by coupling of radicals, mainly *N*- and *para*-forms, and elimination of a proton to give the dimer which then undergoes oxidation on the electrode surface along with aniline to give oligomers, resulting in the chain propagation. Finally, the radical cation of the oligomer reacts further with the radical cation of aniline to elongate the polymer chain, creating finally a dense, adhesive film at the electrode surface.

### 3.2. Scanning Electron Microscopy (SEM) Analysis

**Figure 2.**
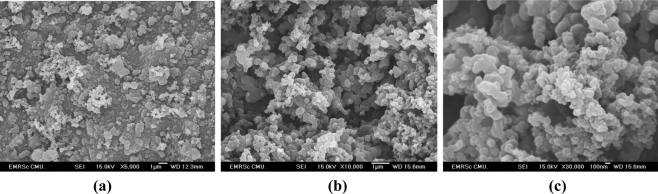
FESEM images of the poly(Ani-*co*-*m*-FcAni) film modified GC electrode with different magnifications at 5 *k*x **(a)**, 10 *k*x **(b)**, and high magnification at 30 *k*x **(c)**.

The surface morphology was investigated by using a field emission scanning electron microscope (FESEM). Figure 2 showed the typical FESEM surface morphology of the poly(Ani-*co-m*-FcAni) with different magnifications. Figure 2(a) shows that the surface of the as-prepared poly(Ani-*co-m*-FcAni) was a three-dimensional network. This feature is common in the polyaniline family prepared in the H_2_SO_4_ medium [19,38]. Figure 2(b,c) shows the high-magnification FESEM views of small nanostructured granules with diameters ranging from ∼100–300 nm. These nanostructures tend to agglomerate in the high porosity interconnected network. The electrochemically synthesized poly(Ani-*co-m*-FcAni) film with nanostructures on GCE would significantly activate the electrode surface and accelerate the electron transfer, and had a high surface area as an ideal electrode material favoring a high performance for electrocatalytic oxidation of AA, as discussed below.

### 3.3. Electrocatalytic Oxidation of AA

The cyclic voltammetric characterization was performed in order to study the electrochemical behavior of the bare GC and poly(Ani-*co*-*m*-FcAni)/GCE towards AA oxidation in 0.1 M citrate buffer (pH 5.0). The overall reaction of ascorbic acid oxidation can be expressed by the following reaction:

(1) $$\underset{\left(\text{ascorbic acid}\right)}{C_{6}H_{8}O_{6}}\;\;\;\;\;\;\;\to \;\;\;\;\;\;\;\;\underset{\left(\text{dehydroascorbate}\right)}{C_{6}H_{6}O_{6}}\;\;\;\;\;\;\;\;+\;\;\;\;\;\;\;\;2H^{+}\;\;\;\;\;\;\;\;+\;\;\;\;\;\;\;\;2e^{-}$$

**Scheme 2.**
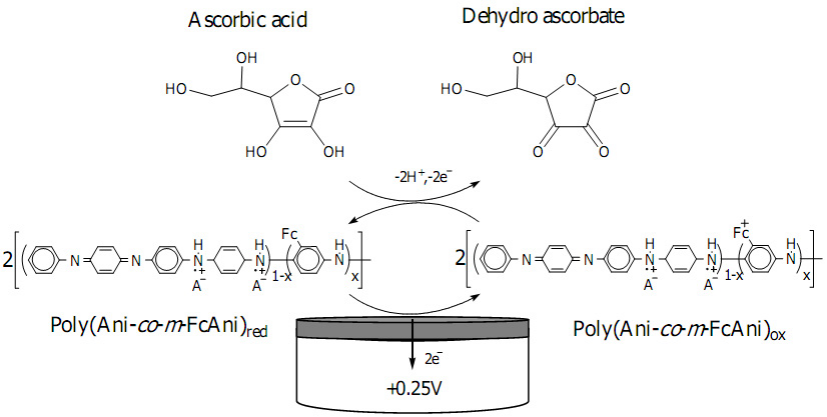
A proposed mechanism for the electrocatalytic oxidation of ascorbic acid to dehydroascorbate at the poly(Ani-*co*-*m*-FcAni) film modified GCE.

A proposed mechanism for the electrocatalytic oxidation of ascorbic acid to dehydroascorbate at poly(Ani-*co*-*m*-FcAni)/GCE is seen in Scheme 2.

**Figure 3.**
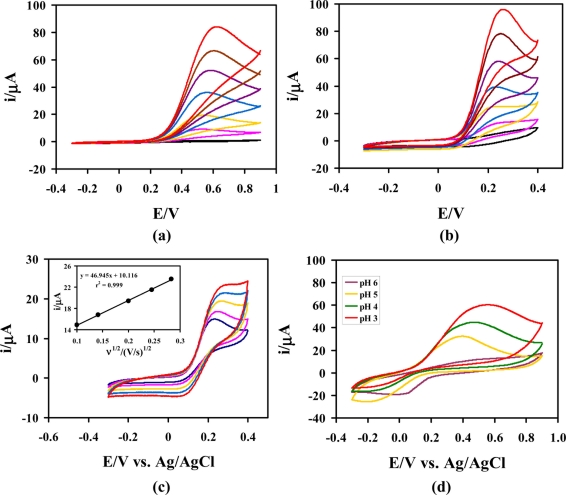
The CVs of AA at **(a)** the bare GCE and at **(b)** the poly(Ani-*co*-*m*-FcAni)/GCE in 0.1 M citrate buffer (pH 5.0) at scan rate of 50 mV s^−1^ in the presence of AA at concentrations: 0.0, 1.0, 2.0, 4.0, 6.0, 8.0 and 10.0 mM (*vs.* Ag/AgCl). **(c)** CVs with various potential scan rates in the presence of 2.0 mM AA in 0.1 M citrate buffer (pH 5.0) at the poly(Ani-*co*-*m*-FcAni)/GCE. Inset shows the relationship between the oxidation current and the square root of the scan rate with linear regression, and **(d)** CVs of 4.0 mM AA at the poly(Ani-*co*-*m*-FcAni)/GCE at different pH buffer solutions.

The oxidation of AA at the unmodified GCE typically requires undesirably high working potentials (≥+0.5 V *vs.* Ag/AgCl). To compare the AA oxidation at both unmodified and modified electrodes. CVs were recorded at a bare GC and poly(Ani-*co*-*m*-FcAni)/GCEs. Figure 3(a) shows that the anodic peak potentials (*E*_p,a_) of AA at the bare GCE were located at about +0.55 V (*vs.* Ag/AgCl) in 0.1 M citrate buffer (pH 5.0). Electrochemical oxidation of AA on the poly(Ani-*co*-*m*-FcAni)/GCE was investigated under the same conditions. Figure 3(b) shows that the *E*_p,a_ of AA at the poly(Ani-*co*-*m*-FcAni)/GCE was located at about +0.25 V (*vs.* Ag/AgCl). The anodic peak currents increased with increasing the concentration of AA. Only oxidation peaks were observed at both of bare and the GCE-modified electrode in the CVs. The results from Figure 3(a,b) shows clearly that the GCE modified with poly(Ani-*co*-*m*-FcAni) film gave a much lower AA peak potential than the unmodified GCE, due to its participation in the AA oxidation reaction. Compared with the unmodified GCE under the same conditions, the higher currents for the oxidation of AA at the modified GCE were attributed to the presence of poly(Ani-*co*-*m*-FcAni) on the surface of electrode. These currents were higher than that on the bare GCE, and the oxidation potential of AA at the poly(Ani-*co*-*m*-FcAni) film modified GCE was also lower about 0.3 V than that on the bare GCE in 0.1 M citrate buffer (pH 5.0). Thus, these experimental results confirmed that the modified GCE with poly(Ani-*co*-*m*-FcAni) film effectively catalyzed the oxidation of AA, and was expected to provide a better electrocatalysis for AA oxidation than the bare GCE.

To study the effect of scan rate on electrocatalytic properties towards AA oxidation. CVs were recorded at the poly(Ani-*co*-*m*-FcAni)/GCE at different scan rates in 0.1 M citrate buffer (pH 5.0) as shown in Figure 3(c). As can be seen, the increase in potential scan rate induced a corresponding increase in peak current and resulted in a shift to more positive values for the electrocatalytic oxidation of AA. The shift of the peak potential was observed as an irreversible electrochemical reaction and a kinetic limitation in the reaction between the redox sites of poly(Ani-*co*-*m*-FcAni) and AA. This indicates that the electrocatalytic oxidation at poly(Ani-*co*-*m*-FcAni) modified GCE was a surface-controlled electrochemical reaction and diffusion-controlled electrode process of AA. The peak current varies linearly with the square root of the scan rate. The linear equation between peak currents and the square root of the scan rate is presented as follows: I_AA_ (μA) = 46.9450υ (V/s)^1/2^ + 10.1160 with a linear relative correlation coefficient of 0.999, indicating that regression line is very well fitted with experimental data. Thus, these results demonstrated that the poly(Ani-*co*-*m*-FcAni)/GCE can be used for the determination of AA.

The effect of pH to the electrocatalytic oxidation of AA at the poly(Ani-*co*-*m*-FcAni)/GCE was investigated with different pH buffer solutions containing 4.0 mM AA using cyclic voltammetry as shown in Figure 3(d). The peak current from the electrocatalytic oxidation of AA in acid solution was higher than that in basic solution. Due to the instability of AA in basic solution, the catalytic current decreased at higher pH. The peak potentials shifted towards negative potential with increasing solution pH between 3 and 6. According to the results obtained from CVs at various pH values, the pH 5 was chosen as the best for the supporting solution for the further investigation.

### 3.4. Effect of the Applied Potential and Scan Cycle

**Figure 4.**
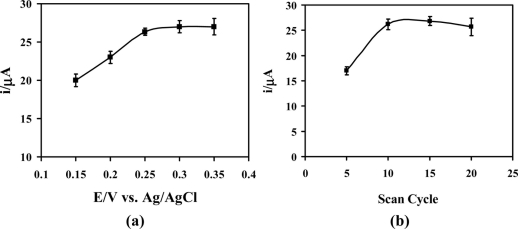
The optimization of the applied potential **(a)** and scan number **(b)** for electrocatalytic oxidation of AA at the poly(Ani-*co*-*m*-FcAni)/GCE in 0.1 M citrate buffer (pH 5.0) at scan rate of 50 mV s^−1^ (n = 3).

In order to optimize the experimental conditions for measurement of AA oxidation at the poly(Ani-*co*-*m*-FcAni)/GCE, the effect of the applied potential and scan cycle used for synthesis of the poly(Ani-*co*-*m*-FcAni) was investigated. Figure 4(a) shows the effect of applied potential at the poly(Ani-*co*-*m*-FcAni)/GCE for electrocatalytic oxidation of AA. It has been clearly shown that the poly(Ani-*co*-*m*-FcAni)/GCE can catalyze AA by applying different potentials in the range from +0.15 to +0.35 V to the working electrode while immersed in the presence of 0.1 M citrate buffer (pH 5.0) containing 2.0 mM AA. As can be seen, the signals obtained were very reproducible. At the higher potential applied, an easier AA oxidation process occurred; however, increasing applied potential typically increases the background current responses from the poly(Ani-*co*-*m*-FcAni) film [12]. Thus, the most appropriate potential of +0.25 V was selected as the applied potential for further studies of AA electrocatalytic oxidation.

Cyclic votammetry is a simple and rapid technique for controlling the thickness of the polymer films. By altering the scan number in electrochemical polymerization, poly(Ani-*co*-*m*-FcAni) films of different thickness were obtained. The electrochemical polymerization of poly(Ani-*co*-*m*-FcAni) at the electrode surface was examined for different numbers by cycling in the range from 5 to 20 scan cycles. Figure 4(b) shows the current of 2.0 mM AA in 0.1 M citrate buffer (pH 5.0) when using GCE-modified with different scan cycles for the synthesized of poly(Ani-*co*-*m*-FcAni)/GCEs. As can be seen, the lowest oxidation current was observed at the 5th-scan cycle. The oxidation current increased upon increasing the scan cycle from 5 to 10 and reached a plateau after that. For the 15th and 20th scan numbers, no enhancement appeared. It could be assumed that a thicker poly(Ani-*co*-*m*-FcAni) film was obtained, resulting a lower catalytic performance [12]. Therefore, the scan number at 10th cycle was selected as an optimum scan cycle in electrochemical polymerization of poly(Ani-*co*-*m*-FcAni) modified GCE for further detailed studies.

### 3.5. Amperometric Measurement

**Figure 5.**
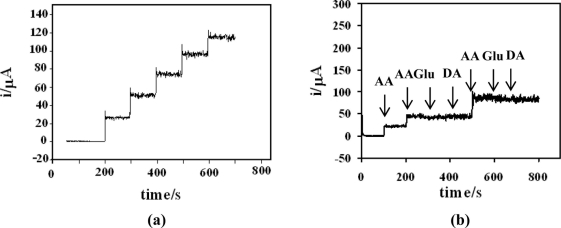
**(a)** Typical amperometric *i*-*t* curve of poly(Ani-*co*-*m*-FcAni)/GCE to successive additions of 0.1 mM AA into a stirred system of 0.1 M citrate buffer (pH 5.0) at +0.25 V. **(b)** Amperometric response for interferences recorded using poly(Ani-*co*-*m*-FcAni)/GCE upon successive additions of 0.1 mM AA, 1.0 mM DA and 1.0 mM Glu.

Figure 5(a) displays the typical amperometric response of the poly(Ani-*co*-*m*-FcAni)/GCE to AA. The amperometric study was carried out through the successive addition of AA into a continuously stirred batch system of 0.1 M citrate buffer (pH 5.0). At the applied potential of +0.25 V (*vs.* Ag/AgCl), the oxidative current increased with increasing concentrations of AA. A linear relationship between oxidation current and concentration of AA was observed in the range from 0.05 × 10^−3^ to 5.7 × 10^−3^ M. Linear calibration was obtained, with a coefficient of 0.997, demonstrating the good relationship between oxidation current and concentration. The limit of detection (LOD) was estimated to be 2.0 μM based on the signal-to-noise ratio (S/N = 3). These experimental results indicated that the poly(Ani-*co*-*m*-FcAni)/GCE has potential application as a chemical sensor for the determination of AA.

In biological and soft drink samples, glucose (Glu) and dopamine (DA) are strong interferents that are electrochemically oxidized at almost the same potential as AA. Figure 5(b) shows the amperometric responses for AA, Glu and DA at the poly(Ani-*co*-*m*-FcAni)/GCE. The successive additions of the same concentration of 0.1 mM AA, 1.0 mM DA and 1.0 mM Glu were investigated by applying potential at +0.25 V (*vs.* Ag/AgCl). It can be seen obviously that additional signals to the current response of AA were not observed due to the successive additions of Glu and DA. Thus, this modified electrode could be applied as the chemical sensor for a practical determination of AA in the presence of Glu and DA. Other electrochemical experiments were then carried out to study the effects of foreign species such as glucose, fructose, sucrose, galactose and sodium chloride. Each interference study was carried out at the applied potential of +0.25 V (*vs.* Ag/AgCl) in 0.1 M citrate buffer (pH 5.0) containing 0.1 mM AA. The amperometric measurements can tolerate 200-fold excesses of three interfering species, namely glucose fructose and sucrose, 240-fold of sodium chloride, and 300 fold of galactose.

## 4. Conclusions

In conclusion, a chemically modified GCE based on poly(Ani-*co*-*m*-FcAni) was successfully prepared by electrochemical copolymerization in 0.5 M H_2_SO_4_ containing 30% ACN, 0.1 M Ani and 0.005 M *m*-FcAni. FESEM images showed that the poly(Ani-*co*-*m*-FcAni) film was deposited on the surface of GCE. From electrochemical experiments, the CVs showed that the *E*_pa_ of AA was shifted from +0.55 V at bare GCE to +0.25 V at the poly(Ani-*co*-*m*-FcAni)/GCE with a greatly enhanced current response. The 10th cycle scan number was selected as an optimum one in the electrochemical polymerization. The amperometric responses demonstrated an excellent selectivity for AA determination over Glu and DA. Thus, this poly(Ani-*co*-*m*-FcAni)/GCE could be applied as an amperometric sensor for the selective detection of AA in biological and soft drink samples.
